# Floral organ development goes live

**DOI:** 10.1093/jxb/eraa038

**Published:** 2020-01-23

**Authors:** Léa Rambaud-Lavigne, Angela Hay

**Affiliations:** 1 Max Planck Institute for Plant Breeding Research, Carl-von-Linné-Weg, Köln, Germany; 2 Trinity College Dublin, Ireland

**Keywords:** APETALA1, *Arabidopsis thaliana*, ATML1, *Cardamine hirsuta*, floral organ number, live-cell imaging, petal, robustness, sepal, SUPERMAN

## Abstract

The chance to watch floral organs develop live is not to be missed! Here, we outline reasons why quantitative, live-cell imaging is an important approach to study floral morphogenesis, and provide a basic workflow of how to get started. We highlight key advances in morphodynamics of lateral organ development, and discuss recent work that uses live confocal imaging to address the regulation of floral organ number, its robustness, and patterning mechanisms that exploit stochasticity.

## Teaser

Quantitative live-cell imaging is an exciting approach to study floral organs. In this review, we highlight recent advances using this approach to understand floral organ initiation and cell fate acquisition.

## Introduction

Morphogenesis is the fascinating process by which a handful of cells develop into complex, three-dimensional forms. Floral organs have some of the most diverse and complex forms found in plants. Research over the past three decades has elucidated many of the gene products and molecular mechanisms that control the unique identities of different floral organs. Moreover, it was already appreciated three decades ago that many, though certainly not all, of these gene functions are evolutionarily conserved (Coen and Meyerowitz, 1991; Causier *et al.*, 2010). A major challenge now is to relate these gene activities to the growth of floral organs to understand what Enrico Coen calls ‘the genetics of geometry’ (Coen *et al.*, 2004).

**Fig. 1.**
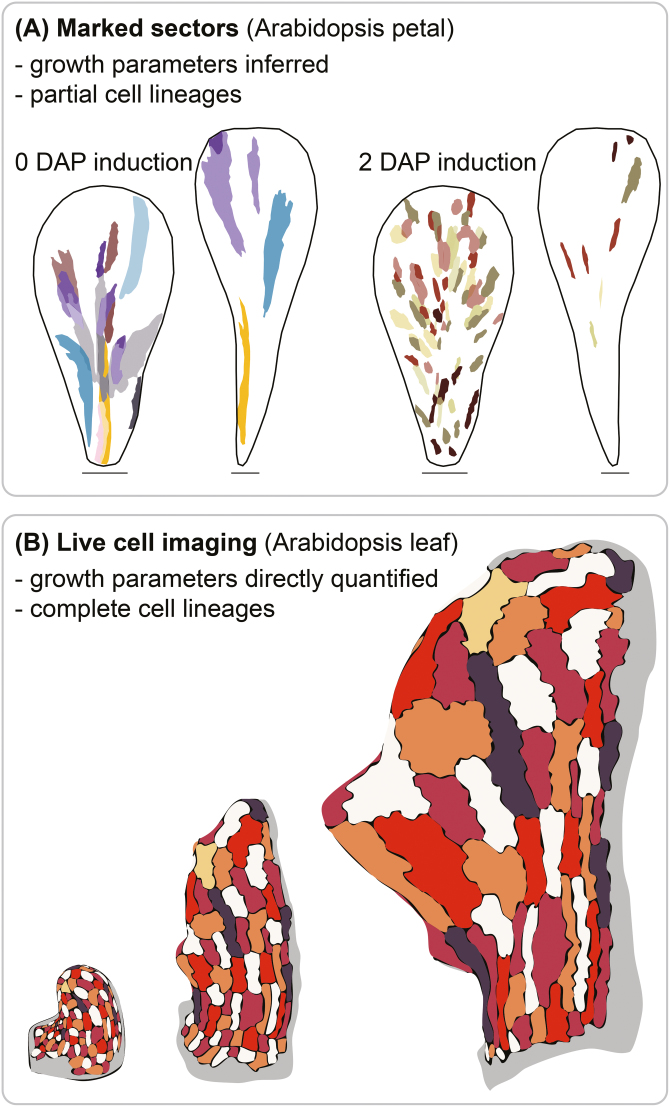
Lineage tracing by sector analysis or live imaging. (A) Marked sectors can be induced and visualized at a range of developmental stages in order to relate growth to organ shape. For example, sectors induced at 0 days after pollination (DAP) in Arabidopsis flowers and visualized in petals at 6 DAP (left) and 12 DAP (right) are larger than sectors induced at 2 DAP and visualized in 6 DAP (left) and 12 DAP (right) petals [cartoons show independent sectors from multiple flowers superimposed on average petal shapes; scale bars=100 μm (6 DAP), 300 μm (12 DAP) (Sauret-Güeto *et al.*, 2013)]. This method infers growth parameters from analysing sector size, shape, and orientation. This method does not usually recover all cell lineages that produce an organ. (B) Live imaging follows a growing organ, at cellular resolution, over several time points, such that complete cell lineage patterns can be mapped for a single sample. For example, cartoons show cell lineage patterns in a single Arabidopsis leaf at three successive stages of development (Kierzkowski *et al.*, 2019). Growth parameters can be directly quantified, rather than inferred, from these cell lineage patterns.

**Fig. 2.**
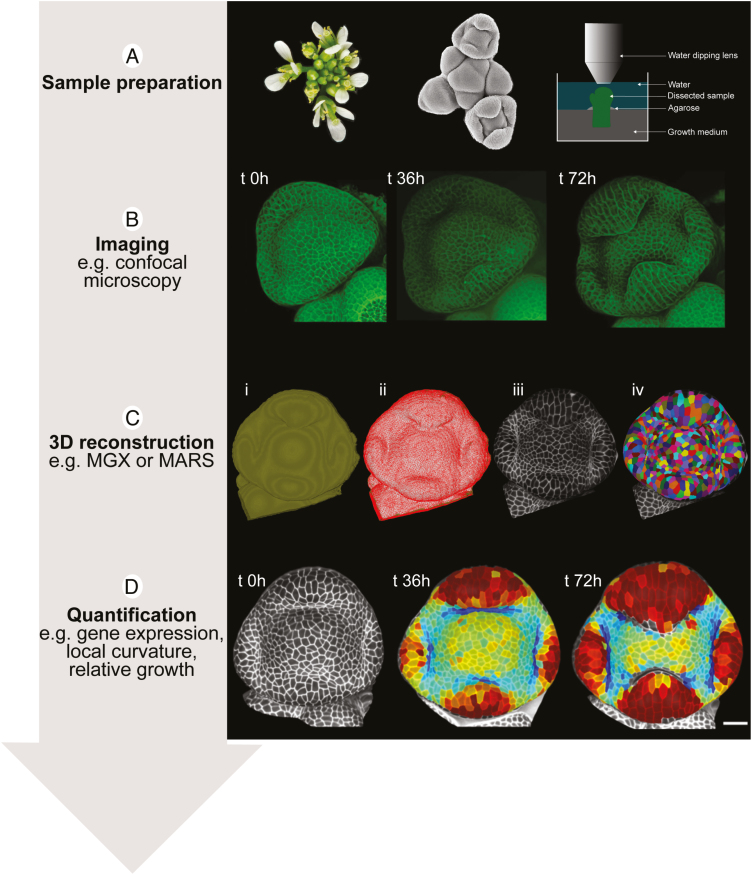
Step-by-step workflow for quantitative live imaging of floral organ development (in *Cardamine hirsuta*). (A) Prepare the sample by removing flowers (left) to expose floral meristems initiating at the shoot apical meristem (middle). Cut off the dissected apex and place in growth medium, using agarose to support the apex in an upright position. Place the sample in a suitable growth environment to maintain growth and development throughout the experiment. Image the sample with a long working distance, water-dipping lens with a good numerical aperture (right). (B) Image cell outlines (and other fluorescent markers in separate channels) in the same flower at successive intervals during floral organ development to acquire *z*-stacks at each time point (example from McKim *et al.*, 2017). (C) Use software such as MorphoGraphX (MGX) or MARS-ALT to accurately segment cells in confocal *z*-stacks. A typical MGX workflow: (i) load *z*-stack and detect sample surface; (ii) extract curved surface as triangular mesh; (iii) project 3-D image data on to this mesh to create a curved image of the outer layer of cells; (iv) segment cells. (D) Track cell lineages across multiple time points to quantify variables of interest, such as growth or gene expression, which can be represented as heat maps (example growth maps from McKim *et al.*, 2017).

This task involves a more quantitative approach to the long-standing genotype to phenotype problem. To describe shape transitions in different genotypes requires quantitative, metric descriptions of growth patterns. To understand how growth influences and responds to the action of genes requires a quantitative framework. The problem of describing and understanding growth quantitatively has become a main topic of multidisciplinary research (Goriely, 2017). Petals and leaves have been a study system of choice to approach these questions, and cell lineage tracing has been one of the most important tools to quantify the parameters underlying growth of these organs (Poethig, 1987) (Fig. 1). By genetically marking dividing cells, their mitotic descendants can be identified as clonal sectors. Cells can be marked using endogenous transposons, such as the temperature-sensitive Tam*3* transposon in snapdragon (Rolland-Lagan *et al.*, 2003; Green *et al.*, 2010), or by transgenic approaches, such as using a heat shock-inducible Cre–Lox system, in Arabidopsis (Sauret-Güeto *et al.*, 2013) (Fig. 1A). Some key growth parameters can then be inferred by analysing the resulting clone patterns, for example the rate and duration of growth, the degree of anisotropy, and the main direction of growth (Rolland-Lagan *et al.*, 2003; Green *et al.*, 2010; Sauret-Güeto *et al.*, 2013). Although you cannot control which individual cells will be marked, the ease of generating and analysing thousands of sectors makes this a robust technique to estimate growth. One caveat to inferring growth parameters by this method is that the geometry of the initial cell influences the size and shape of the resulting sector. For example, anisotropic growth is inferred from elongated sectors, but an elongated cell will produce an elongated sector even if growth is isotropic. These issues can be avoided by observing individual cell lineages in a growing organ over time, and directly measuring the parameters that describe its growth (Fig. 1B). This can be achieved by live (time-lapse) imaging and subsequent segmentation of cells from each time point as described in Fig. 2. New developments in imaging and software have accelerated this approach in recent years, allowing key advances to be made in plant morphodynamics (Box 1).

Box 1. **Key developments in live-cell imaging of lateral organ morphogenesis**

A recent study from Kierzkowski *et al.* (2019) shows the current state-of-the-art in using live-cell imaging to understand how genes shape diversity. The authors follow a morphodynamic approach, combining live-cell imaging and genetics with quantitative image analysis and computational modelling, to explain how the interplay between growth and patterning generates leaf shape diversity. Other studies have combined live-cell imaging with modelling to study lateral organ morphogenesis (e.g. Kuchen *et al.*, 2012; Hervieux *et al.*, 2016, 2017; Hong *et al.*, 2016; Fox *et al.*, 2018; Ripoll *et al.*, 2019), but this study presents the following key developments:

- **Complete cellular growth and fate maps.** Quantitative data on cellular growth at high spatial and temporal resolution were produced for multiple genotypes using live-cell imaging of the complete leaf surface, including the margin, every 24 h for eight consecutive days after primordium initiation.
- ** Growth alignment graphs.** By aligning cells based on organ-wide positional coordinates, the contribution of cellular growth properties to final organ shape was accurately compared at equivalent positions between samples.
- ** Comparative genetics.** Parallel genetic studies in two related species that differ in leaf shape (*A. thaliana*, simple; *C. hirsuta*, dissected) were used to produce a growth-based framework that explains not only development but also diversity of leaf shape.
- ** Computational modelling.** The finite element method was used to simulate growth and patterning in physically connected tissues, while a geometric model was used to explore geometries produced by interactions between local growth inputs with an organ-wide growth field.
- ** Trait reconstruction.** Model predictions were validated by transgenic reconstruction of *C. hirsuta* leaf shape in *A. thaliana*.

**Figure:**
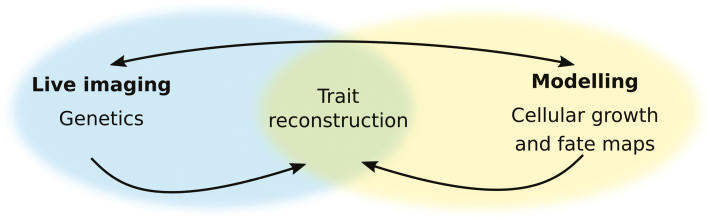

Live-cell imaging, where growing plants are imaged at the cellular level repeatedly over time, can be used to address many developmental questions, as we will highlight further in this review. However, the interactions that link growth patterns to gene activities are often difficult to interpret without considering the mechanics of growth. This is because genetically specified growth of a region, such as growth rate and anisotropy, occurs within the mechanical constraints of its neighbouring regions. Therefore, the resultant growth includes the tissue deformations that arise due to this connectivity (Coen and Rebocho, 2016; Rebocho *et al.*, 2017). Computational modelling offers a way to navigate through these issues by incorporating interactions between the main direction of growth, regional identities, and mechanics in a growing framework (Kennaway *et al.*, 2011). Early results from working in this type of quantitative framework showed, for example, that the main direction of growth is important to generate the asymmetry that characterizes the dorsal petal lobe in snapdragon (Rolland-Lagan *et al.*, 2003). Analysing the entire snapdragon corolla and the Arabidopsis petal gave further indications that genes control petal shape by independently specifying the properties of regional growth and also overall growth directions in the petal (Green *et al.*, 2010; Sauret-Güeto *et al.*, 2013). More recent advances have come from investigating how genes control leaf shape (Kuchen *et al.*, 2012; Kierzkowski *et al.*, 2019; Whitewoods *et al.*, 2020). By using quantitative live imaging, comparative experimental systems, and mechanistic models, Kierzkowski *et al.* (2019) showed, for example, that differences between *Arabidopsis thaliana* and *Cardamine hirsuta* leaf shape result from local modification of growth during patterning and a global redistribution of growth that results from delayed differentiation (Box 1).

So, given the advantages of live-cell imaging, what do you need to know to get started?

## Going live

A typical workflow for quantitative, live-cell imaging of floral organs is illustrated in Fig. 2. First, the shoot apex is dissected to expose the youngest floral primordia of interest (Fig. 2A). The apex is secured in position for the duration of the experiment and can be cut from the plant and cultured on growth medium to facilitate viewing (Prunet *et al.*, 2016). Whether the sample is left intact or cultured, it is critical to compare its overall growth and development with those of control plants to understand whether the experiment causes any deviations, for example by timing the progression of floral primordia through consecutive developmental stages (Smyth *et al.*, 1990). Confocal laser scanning microscopy is commonly used to image the live sample at cellular resolution over several time points that best fit the developmental process of interest (Fig. 2B). For example, samples are often imaged at 24 h time intervals over 2–5 d (McKim *et al.*, 2017; Prunet *et al.*, 2017; Monniaux *et al.*, 2018; Ripoll *et al.*, 2019) or up to 7–8 d (Hervieux *et al.*, 2016; Kierzkowski *et al.*, 2019), or at shorter 8–12 h time intervals (Hong *et al.*, 2016; Caggiano *et al.*, 2017; Meyer *et al.*, 2017). These image stacks, comprising 3-D volumetric data, can then be processed with software, such as MorphoGraphX or MARS-ALT, to extract the sample contour and accurately segment the cells (Fernandez *et al.*, 2010; Barbier de Reuille *et al.*, 2015) (Fig. 2C). Because morphogenesis involves 3-D deformations, it is important to quantify each image as 3-D data or to summarize the 3-D data as a curved surface image. For example, MorphoGraphX can be used to extract the shape of a sample as a mesh (Fig. 2Ci–ii), and project on to this mesh the 3-D image data just below the surface, creating a curved image of the outer layer of cells (Fig. 2Ciii). This allows cell outlines to be accurately extracted without the distortions associated with a flat 2-D projection (Barbier de Reuille *et al.*, 2015). The resulting curved surface images can then be segmented into individual cells (Fig. 2Civ). Comparing segmentation files at successive time points accurately maps the cell lineage patterns (Strauss *et al.*, 2019). Various features, such as gene expression, amount of growth, and growth direction, can then be quantified at a cellular level over time and displayed as heat maps on curved surface images. Further information to get started with computational image processing in microscopy, with an emphasis on the Fiji image analysis platform, can be found in the detailed tutorial by Roeder (2019).

In the next sections, we will discuss work over the past 2 years that has used live imaging of floral organ development to shed light on the regulation of organ number and robustness.

## Floral organ number and robustness

Floral organs acquire their distinct identities according to the ABC model of flower development (Coen and Meyerowitz, 1991). However, less is known about how the flower is partitioned into distinct whorls containing the correct number of floral organs. SUPERMAN (SUP) is a transcriptional repressor that specifies the boundary between stamens and carpels. Flowers of *sup* mutants form too many stamens at the expense of carpels. However, until recently, it had been impossible to distinguish whether these extra stamens originate from an organ identity change in whorl 4 or the overproliferation of whorl 3. Prunet *et al.* (2017) tackled this question using live confocal imaging. They showed unequivocally that extra stamens in *sup* flowers arise from whorl 4 cells that change identity from carpel to stamen. Extended proliferation of stem cells, rather than whorl 3 cells, in *sup* flowers allows the formation of further stamens (Prunet *et al.*, 2017). Key to these results was the ability to track SUP and whorl 3 markers [APETALA3 (AP3) and PISTILLATA] at high spatial and temporal resolution in single samples. By analysing the overlap between SUP and AP3, it was clear that SUP accumulates not only in whorl 3 as previously thought, but also in whorl 4. Live imaging of *sup* mutant flowers showed that non-*AP3*-expressing cells in whorl 4 switched identity from carpel to stamen by starting to express *AP3* (Prunet *et al.*, 2017). Therefore, SUP acts in these whorl 4 cells, adjacent to the boundary with whorl 3, to repress *AP3* expression and partition stamen and carpel developmental programmes in adjacent organs.

**Fig. 3.**
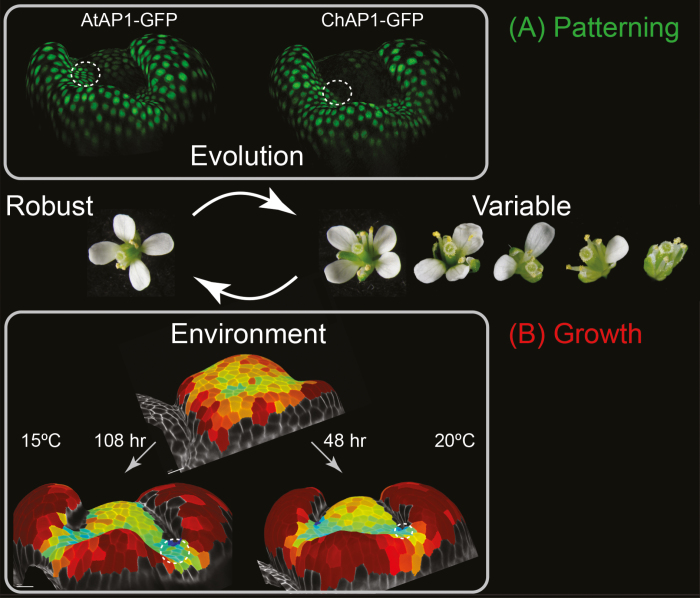
Petal number robustness. Brassicaceae flowers, such as Arabidopsis, usually have a robust petal number of four. *C. hirsuta* flowers lack this robustness and, instead, have a variable number of petals between zero and four. (A) *APETALA1* (*AP1*) divergence underlies this evolutionary transition (Monniaux *et al.*, 2018). Live imaging and projection of AP1 signal on the curved surface of *C. hirsuta* flowers showed that *A. thaliana* AP1 (AtAP1–GFP) was expressed in more cells than *C. hirsuta* AP1 (ChAP1–GFP) in the small regions where petals initiate on the floral meristem (dashed circles). (B) Growth differences underlie the plasticity of *C. hirsuta* petal number in response to environment. Petal number is more robust at cooler ambient temperature of 15 °C, and more variable at 20 °C (McKim *et al.*, 2017). Live imaging and growth analysis of cell lineages on the curved surface of *C. hirsuta* flowers showed that floral meristem maturation was delayed at 15 °C (108 h) relative to 20 °C (48 h). Because the floral meristem grew for longer at 15 °C, relative to sepals, more space was produced for petals to initiate between sepals (dashed circles). Therefore, patterning differences underlie evolutionary transitions in petal number robustness, while growth differences alter petal number robustness in response to environment.

Although the number of floral organs is typically robust in *A. thaliana* flowers, the same is not true for its close relative, *C. hirsuta*. In *C. hirsuta* flowers, petal number varies between zero and four (Fig. 3A). Is this because petals fail to initiate or fail to grow in *C. hirsuta*? Monniaux *et al.* (2018) addressed this question using live confocal imaging. Petal formation is directed by local accumulation of auxin, and DR5 reporters can be used to mark sites of transcriptional auxin response during petal formation (Lampugnani *et al.*, 2013). However, these peaks of auxin activity are transitory and mark only a few cells at the sites of petal initiation. So, live imaging helps ‘catch’ these events. By mapping growth in the same samples, it was clear that DR5 expression preceded growth of floral meristem cells at petal initiation sites in *A. thaliana* (Monniaux *et al.*, 2018). Yet, in contrast to *A. thaliana*, petal initiation sites on the floral meristem in *C. hirsuta* were often not marked by DR5 expression (Monniaux *et al.*, 2018). Therefore, the number of petals in a *C. hirsuta* flower is affected by variation in organ initiation rather than growth. Monniaux *et al.* went on to show that regulatory divergence in the APETALA1 (AP1) MADS-box transcription factor can account for the species-specific difference in petal number robustness between *A. thaliana* and *C. hirsuta* (Monniaux *et al.*, 2018). When swapped into *C. hirsuta*, the *A. thaliana* copy of *AP1* was expressed in a larger domain of cells in the petal whorl than *C. hirsuta AP1*, resulting in robust petal number (Fig. 3A). What is still unclear is the link between *AP1* divergence and destabilization of the patterning of auxin peaks that are required for petal organogenesis.

Loss of robustness makes *C. hirsuta* petal number sensitive to genetic, environmental, and stochastic changes (Monniaux *et al.*, 2016; Pieper *et al.*, 2016; McKim *et al.*, 2017). As such, petal number is strongly influenced by natural genetic variation, but also varies in response to seasonal cues, such as day length, winter cold, and, in particular, ambient temperature. In the field, spring-flowering plants produce more petals and, in the greenhouse, petal number is increased by conditions experienced in spring, such as cool ambient temperature (McKim *et al.*, 2017). McKim *et al.* used live confocal imaging to show that cool ambient temperature increases petal number via slowing growth and maturation of the floral bud (McKim *et al.*, 2017). Extending the duration of floral meristem growth at 15 °C versus 20 °C produced larger flat regions between sepals with more space available for petal initiation (Fig. 3B). Therefore, the influence of the environment on floral meristem growth and maturation provides a developmental route for petal number to respond plastically to seasonal conditions in *C. hirsuta*.

Organ development can be robust but still utilize stochastic changes in gene expression to initiate cellular patterning. Sepals have a scattered pattern of highly endoreduplicated giant cells interspersed between smaller cells on their abaxial surface (Roeder *et al.*, 2010). The *A. thaliana* class IV HD-ZIP transcription factor MERISTEM LAYER1 (ATML1) is necessary and sufficient for the formation of giant cells (Roeder *et al.*, 2012; Meyer *et al.*, 2017). However, only a subset of epidermal cells expressing *ATML1* become giant. This raises the question: how does ATML1 initiate giant cell patterning? Meyer *et al.* tackled this question using live confocal imaging. They found that fluctuations in ATML1 concentration produce asymmetries between cells that are read out at G_2_ in the cell cycle as patterning information (Meyer *et al.*, 2017). Key to this result was the use of live imaging to record dynamic patterns of *pATML1::mCitrine-ATML1* gene expression. Using a genetic dosage series, the authors had shown that the proportion of giant cells was sensitive to ATML1 levels (Meyer *et al.*, 2017). However, only by quantifying ATML1 levels in the sepal epidermis over time could they show that protein levels fluctuate in and between each cell. Using nuclear area as a proxy for cell cycle stage, it became clear that peak concentrations of ATML1 during G_2_ could accurately predict cell fate (Meyer *et al.*, 2017). Therefore, cell-autonomous fluctuations generate concentration differences of ATML1 that determine the proportion of giant cells.

## Future perspectives

### Live imaging—what is it good for?

In this review, we have highlighted recent advances in understanding floral organ initiation and cell fate acquisition that were made using quantitative, live-cell imaging. Moreover, this approach has advanced our understanding of lateral organ development more broadly. This is because the comparative expression of multiple genes at high spatial and temporal resolution can help answer questions that are otherwise difficult to resolve. For example, Prunet *et al.* (2017) defined the precise expression domain of SUP by analysing cell-level overlap with AP3, and directly observed cell fate switching in *sup* mutants by following AP3 expression in individual cells over time. In another example, Caggiano *et al.* (2017) simultaneously imaged auxin and organ polarity genes in the shoot apical meristem to show that sites of lateral organ initiation, marked by PINFORMED1, occurred in a narrow ‘gap’ between KANADI1 and REVOLUTA expression domains. Relating gene activities, as studied in real time, to growth can answer additional questions. For example, Kierzkowski *et al.* (2019) resolved how CUP-SHAPED COTYLEDONS2 can both stimulate and repress growth in the leaf margin: first, it triggers auxin activity maxima to promote growth; then, it is restricted to the regions flanking these maxima where it represses growth (Box 1). In another example, Monniaux *et al.* (2018) showed that an auxin activity maximum precedes growth at sites of petal initiation in Arabidopsis. Quantifying fluctuations in gene expression at cellular resolution can provide further answers. For example, Meyer *et al.* (2017) demonstrated a fluctuation-driven patterning mechanism for cell fate in the Arabidopsis sepal by measuring ATMLI1 levels relative to cell cycle progression in individual cells.

### Live imaging—what’s next?

Microscopes are key to live-cell imaging. All of the studies mentioned in this review used confocal laser scanning microscopes to image cell layers at the plant surface. However, the goal of long-term, minimally invasive imaging of not only surface but also internal tissues requires different microscopes. Two-photon excitation microscopy is one option to achieve deep-tissue imaging with reduced photobleaching (Grossmann *et al.*, 2018). Light sheet fluorescence microscopy is an ideal option for long-term, time-resolved imaging due to its high-speed acquisition rates and low energy sample exposure (Grossmann *et al.*, 2018; Ovečka *et al.*, 2018). Although it is more challenging to image flowers with this technique compared with roots, a recent study used light sheet microscopy to image developing flowers continuously for several days at cellular resolution (Valuchova *et al.*, 2019, preprint).

Quantitative live-cell imaging also relies on accurate cell segmentation. Making this process not only accurate but also user-friendly is an essential task. Novel segmentation algorithms based on modern machine learning concepts, such as convolutional neural networks, have the potential to reduce the manual annotation burden and address problems of 3-D cell segmentation (Wolny *et al.*, 2020, preprint). It is also important to provide these image analysis tools via free, open-source software, such as PlantSeg (Wolny *et al.*, 2020, preprint), ilastik (Berg *et al.*, 2019), or MorphoGraphX (Barbier de Reuille *et al.*, 2015). Mechanical force measurements can also be incorporated into live-cell imaging workflows. For example, MorphoRobotX is an extension of MorphoGraphX that provides control and visualization of cellular force microscopy, which is a non-invasive micro-indentation method used to measure cell stiffness (Majda *et al.*, 2019). Exporting cell geometries directly from software such as MorphoGraphX as templates for computational models also helps to create biologically realistic simulations (Mosca *et al.*, 2017). In summary, we discussed the use of live-cell imaging to study floral organ development in this review, but many questions concerning flowering and flowers can, and already do, benefit from this approach. We anticipate much more flowering research going live in the near future.
